# ERYTHEMA ANNULARE CENTRIFUGUM IN PREGNANCY

**DOI:** 10.4103/0019-5154.60371

**Published:** 2010

**Authors:** Engin Senel, Ayşe Tülin Gulec

**Affiliations:** *From the Department of Dermatology, Çankiri State Hospital, 18200 Çankiri, Turkey.*; 1 *From the Department of Dermatology, Baskent University Faculty of Medicine, Ankara, Turkey. E-mail: enginsenel@enginsenel.com*

Sir,

Erythema annulare centrifugum (EAC), the most common form of gyrate erythemas, was first introduced in 1916 by Darier.[1] EAC has been considered to be related to various etiological factors including infections, immunological disorders, malignancy, drugs and foods. However, no disorders, medications or infectious agents have been convincingly linked to the development of EAC.[1,2]

A 28-year-old woman presented with a one-month history of asymptomatic skin eruption which appeared in the 26^th^ week of her first pregnancy. Her medical history was unremarkable. Dermatological examination disclosed multiple annular plaques with a trailing scale at the inner border of the erythema on her abdomen and extremities [Figure 1]. Direct microscopic examination did not reveal any fungal hyphae. Histopathology showed mild hyperkeratosis, mild focal parakeratosis, spongiosis in the epidermis, and partially well demarcated perivascular lymphocytic infiltrate around the blood vessels in a sleeve like arrangement in the superficial dermis [Figures 2 and 3].

**Figure 1:**
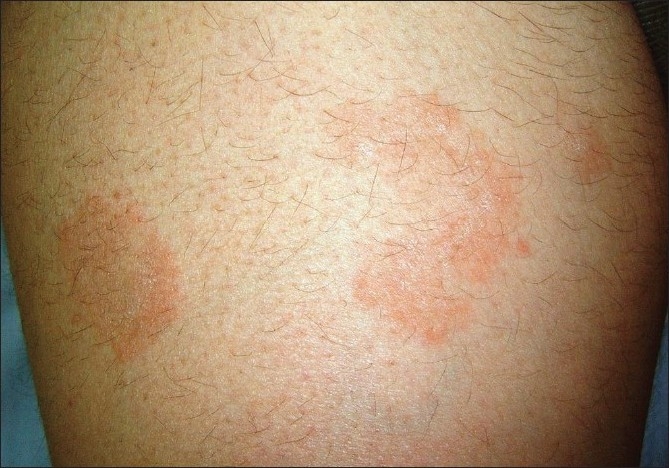
Annular erythematous lesions with the characteristic trailing scale on thigh

**Figure 2:**
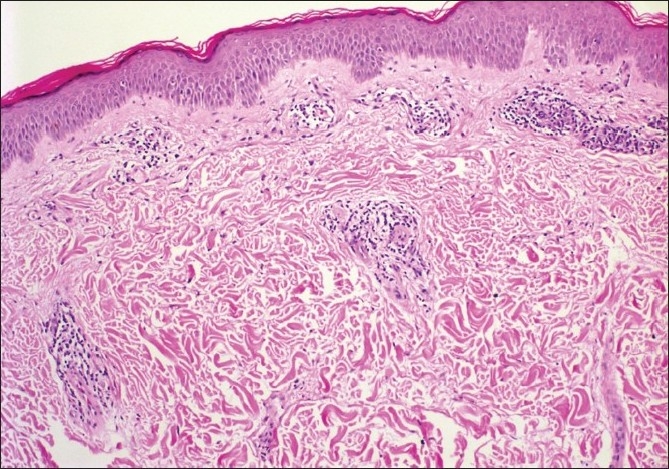
The vessels of the superficial vascular plexuses surrounded by dense lymphocytic infiltrate in a “coat sleeve” pattern (H and E, ×10)

**Figure 3:**
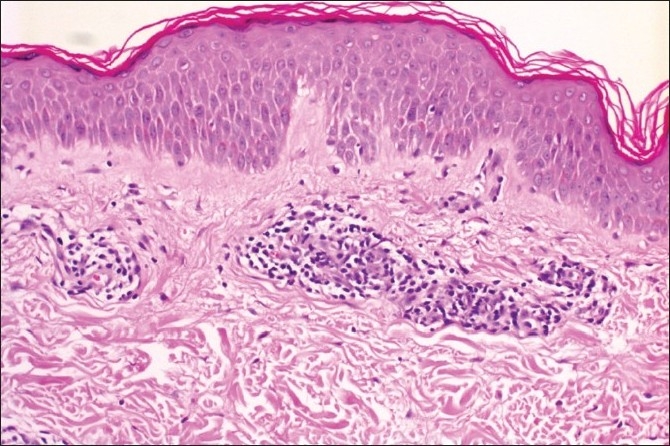
Partially well demarcated perivascular lymphocytic infiltrate (H and E, ×20)

The patient was diagnosed with EAC. Complete blood count, indices of renal, hepatic and thyroid functions, antinuclear antibody test, C-reactive protein, antistreptolysine-O, urinary analysis and stool examination were in normal limits. Since she was pregnant, no therapy was planned. All her lesions resolved gradually in seven weeks. No recurrence was noted during pregnancy and on a four-month follow-up after delivery.

Majority of the cases with EAC are idiopathic.[3] Hormonal changes (elevation of serum estrogen and progesterone levels), which occur during pregnancy might have caused EAC in our patient.

Although EAC is the most common form of the figurate erythemas, there are only two pregnant cases with EAC reported in the literature.[1,2] Rosina *et al*. reported a 28-year-old woman with a two-week history of EAC lesions on her extremities in the 33^rd^ week of her first pregnancy.[1] Choonhakarn and Seramethakun also reported a similar case with EAC lesions on her abdomen and extremities in the 29^th^ week of her first pregnancy.[2] No etiological factor was detected in these patients, and their lesions cleared spontaneously. The authors proposed that increasing estrogen level in pregnancy might be the possible cause of EAC in these patients.[1,2]

Progesterone, a 21-carbon steroid hormone involved in the female menstrual cycle, is also a sine qua non for the establishment and maintenance of pregnancy. The association between EAC and progesterone has been described in the literature. Halevy *et al*. reported a 28-year-old woman with the cyclic annular pruritic lesions aggravated a few days before menstruation in the high progesterone period of the cycle. The authors showed that *in vivo* intradermal progesterone test revealed an immediate-type response and higher IFN-γ release was detected for progesterone in the patient. They suggested that these EAC lesions could be a manifestation of the autoimmune progesterone dermatitis (AIPD).[4]

EAC can occur very rarely in pregnancy and hormonal changes such as significant elevation of estrogen and/or progesterone hormones level may be its possible cause. EAC in pregnancy might be a limited manifestation of AIPD during pregnancy.

## References

[1] Rosina P, D'Onghia FS, Barba A (2002). Erythema annulare centrifugum and pregnancy. Int J Dermatol.

[2] Choonhakarn C, Seramethakun P (1998). Erythema annulare centrifugum associated with pregnancy. Acta Derm Venereol.

[3] Weyers W, Diaz-Cascajo C, Weyers I (2003). Erythema annulare centrifugum: Results of a clinicopathologic study of 73 patients. Am J Dermatopathol.

[4] Halevy S, Cohen AD, Lunenfeld E, Grossman N (2002). Autoimmune progesterone dermatitis manifested as erythema annulare centrifugum: Confirmation of progesterone sensitivity by *in vitro* interferon-gamma release. J Am Acad Dermatol.

